# The Role of Large Language Models in Transforming Emergency Medicine: Scoping Review

**DOI:** 10.2196/53787

**Published:** 2024-05-10

**Authors:** Carl Preiksaitis, Nicholas Ashenburg, Gabrielle Bunney, Andrew Chu, Rana Kabeer, Fran Riley, Ryan Ribeira, Christian Rose

**Affiliations:** 1 Department of Emergency Medicine Stanford University School of Medicine Palo Alto, CA United States

**Keywords:** large language model, LLM, emergency medicine, clinical decision support, workflow efficiency, medical education, artificial intelligence, AI, natural language processing, NLP, AI literacy, ChatGPT, Bard, Pathways Language Model, Med-PaLM, Bidirectional Encoder Representations from Transformers, BERT, generative pretrained transformer, GPT, United States, US, China, scoping review, Preferred Reporting Items for Systematic Reviews and Meta-Analyses, PRISMA, decision support, workflow efficiency, risk, ethics, education, communication, medical training, physician, health literacy, emergency care

## Abstract

**Background:**

Artificial intelligence (AI), more specifically large language models (LLMs), holds significant potential in revolutionizing emergency care delivery by optimizing clinical workflows and enhancing the quality of decision-making. Although enthusiasm for integrating LLMs into emergency medicine (EM) is growing, the existing literature is characterized by a disparate collection of individual studies, conceptual analyses, and preliminary implementations. Given these complexities and gaps in understanding, a cohesive framework is needed to comprehend the existing body of knowledge on the application of LLMs in EM.

**Objective:**

Given the absence of a comprehensive framework for exploring the roles of LLMs in EM, this scoping review aims to systematically map the existing literature on LLMs’ potential applications within EM and identify directions for future research. Addressing this gap will allow for informed advancements in the field.

**Methods:**

Using PRISMA-ScR (Preferred Reporting Items for Systematic Reviews and Meta-Analyses extension for Scoping Reviews) criteria, we searched Ovid MEDLINE, Embase, Web of Science, and Google Scholar for papers published between January 2018 and August 2023 that discussed LLMs’ use in EM. We excluded other forms of AI. A total of 1994 unique titles and abstracts were screened, and each full-text paper was independently reviewed by 2 authors. Data were abstracted independently, and 5 authors performed a collaborative quantitative and qualitative synthesis of the data.

**Results:**

A total of 43 papers were included. Studies were predominantly from 2022 to 2023 and conducted in the United States and China. We uncovered four major themes: (1) clinical decision-making and support was highlighted as a pivotal area, with LLMs playing a substantial role in enhancing patient care, notably through their application in real-time triage, allowing early recognition of patient urgency; (2) efficiency, workflow, and information management demonstrated the capacity of LLMs to significantly boost operational efficiency, particularly through the automation of patient record synthesis, which could reduce administrative burden and enhance patient-centric care; (3) risks, ethics, and transparency were identified as areas of concern, especially regarding the reliability of LLMs’ outputs, and specific studies highlighted the challenges of ensuring unbiased decision-making amidst potentially flawed training data sets, stressing the importance of thorough validation and ethical oversight; and (4) education and communication possibilities included LLMs’ capacity to enrich medical training, such as through using simulated patient interactions that enhance communication skills.

**Conclusions:**

LLMs have the potential to fundamentally transform EM, enhancing clinical decision-making, optimizing workflows, and improving patient outcomes. This review sets the stage for future advancements by identifying key research areas: prospective validation of LLM applications, establishing standards for responsible use, understanding provider and patient perceptions, and improving physicians’ AI literacy. Effective integration of LLMs into EM will require collaborative efforts and thorough evaluation to ensure these technologies can be safely and effectively applied.

## Introduction

### Background

Emergency medicine (EM) is at an inflection point. With increasing patient volumes, decreasing staff availability, and rapidly evolving clinical guidelines, emergency providers are overburdened and burnout is significant [1]. While the role of artificial intelligence (AI) in enhancing emergency care is increasingly recognized, the emergence of large language models (LLMs) offers a novel perspective. Previous reviews have systematically categorized AI applications in EM, focusing on diagnostic-specific and triage-specific branches, emphasizing diagnostic prediction and decision support [2-5]. This review aims to build upon these foundations by exploring the unique potential of LLMs in EM, particularly in areas requiring complex data processing and decision-making under time constraints.

An LLM is a deep learning–based artificial neural network, distinguished from traditional machine learning models by its training on vast amounts of textual data. This enables LLMs to recognize, translate, predict, or generate text or other content [6]. Characterized by transformer architecture and the ability to encode contextual information using several parameters, LLMs allow for nuanced understanding and application across a diverse range of topics. Unlike traditional AI models, which often rely on structured data and predefined algorithms, LLMs are adept at interpreting unstructured text data. This feature makes them particularly useful in tasks such as real-time data interpretation, augmenting clinical decision-making, and enhancing patient engagement in clinical settings. For instance, LLMs can efficiently sift through electronic health records (EHRs) to identify critical patient histories and assist clinicians in interpreting multimodal diagnostic data. In addition, they can serve as advanced decision support tools in differential diagnosis, enhancing the quality of care while reducing the cognitive load and decision fatigue for emergency providers. Furthermore, the content generation ability of LLMs, ranging from technical computer code to essays and poetry, demonstrates their versatility and exceeds the functional scope of traditional machine learning models in terms of content creation and natural language processing.

### Importance

While interest in applying LLMs to EM is gaining momentum, the existing body of literature remains a patchwork of isolated studies, theoretical discussions, and small-scale implementations. Moreover, existing research often focuses on specific use cases, such as diagnostic assistance or triage prioritization, rather than providing a holistic view of how LLMs can be integrated into the EM workflow. Conclusions based on other forms of machine learning are not readily translatable to LLMs. This fragmented landscape makes it challenging for emergency clinicians, who are already burdened by the complexities and pace of their practice, to discern actionable insights or formulate a coherent strategy for adopting these technologies. Despite the promise shown by several models, such as ChatGPT-4 (OpenAI) or Med-PaLM 2 (Google AI), the absence of standardized metrics for evaluating their clinical efficacy, ethical use, and long-term sustainability leaves researchers and clinicians navigating an uncharted territory. Consequently, the potential for LLMs to enhance emergency medical care remains largely untapped and poorly understood.

### Goals of This Review

In light of these complexities and informational disparities, our study undertakes a crucial step to consolidate, assess, and contextualize the fragmented knowledge base surrounding LLMs in EM. Through a scoping review, we aim to establish a foundational understanding of the field’s current standing, from technological capabilities to clinical applications and ethical considerations. This synthesis serves a dual purpose: first, to equip emergency providers with a navigable map of existing research and, second, to identify critical gaps and avenues for future inquiry. As EM increasingly embraces technological solutions for its unique challenges, our goal is to provide clarity to the responsible and effective incorporation of LLMs into clinical practice.

## Methods

### Overview

We adhered to the PRISMA-ScR (Preferred Reporting Items for Systematic Reviews and Meta-Analyses extension for Scoping Reviews) checklist [7] and used the scoping review methodology proposed by Arksey and O’Malley [8] and furthered by Levac et al [9]. This included the following steps: (1) identifying the research question; (2) identifying relevant studies; (3) selecting studies; (4) charting the data; (5) collating, summarizing, and reporting the results; and (6) consultation. Our full review protocol is published elsewhere [10].

### Identifying the Research Question

The overall purpose of this review was to map the current literature describing the potential uses of LLMs in EM and to identify directions for future research. To achieve this goal, we aimed to answer the primary research question: “What are the current and potential uses of LLMs in EM described in the literature?” We chose to explicitly focus on LLMs as this subset of AI is rapidly developing and generating significant interest for potential applications.

### Identifying Relevant Studies

In August 2023, we searched Ovid MEDLINE, Embase, Web of Science, and Google Scholar for potential citations of interest. We limited our search to papers published after January 2018 as the Bidirectional Encoder Representations from Transformers (BERT; Google) model was introduced that year and considered by many to be the first in the contemporary class of LLMs [11]. Our search strategy (Multimedia Appendix 1), created in consultation with a medical librarian, combined keywords and MeSH (Medical Subject Headings) terms related to LLMs and EM. We reviewed the bibliographies of identified studies for potential missed papers.

### Study Selection

Citations were managed using Covidence web-based software (Veritas Health Innovation). Manuscripts were included if they discussed the use of an LLM in EM, including applications in the emergency department (ED) and prehospital and periadmission settings. Furthermore, we included use cases related to public health, disease monitoring, or disaster preparedness as these are relevant to EDs. We excluded studies that used other forms of machine learning or natural language processing that were not LLMs and studies that did not clearly relate to EM. We also excluded cases where the only use of an LLM was in generating the manuscript without any additional commentary.

**Figure 1:**
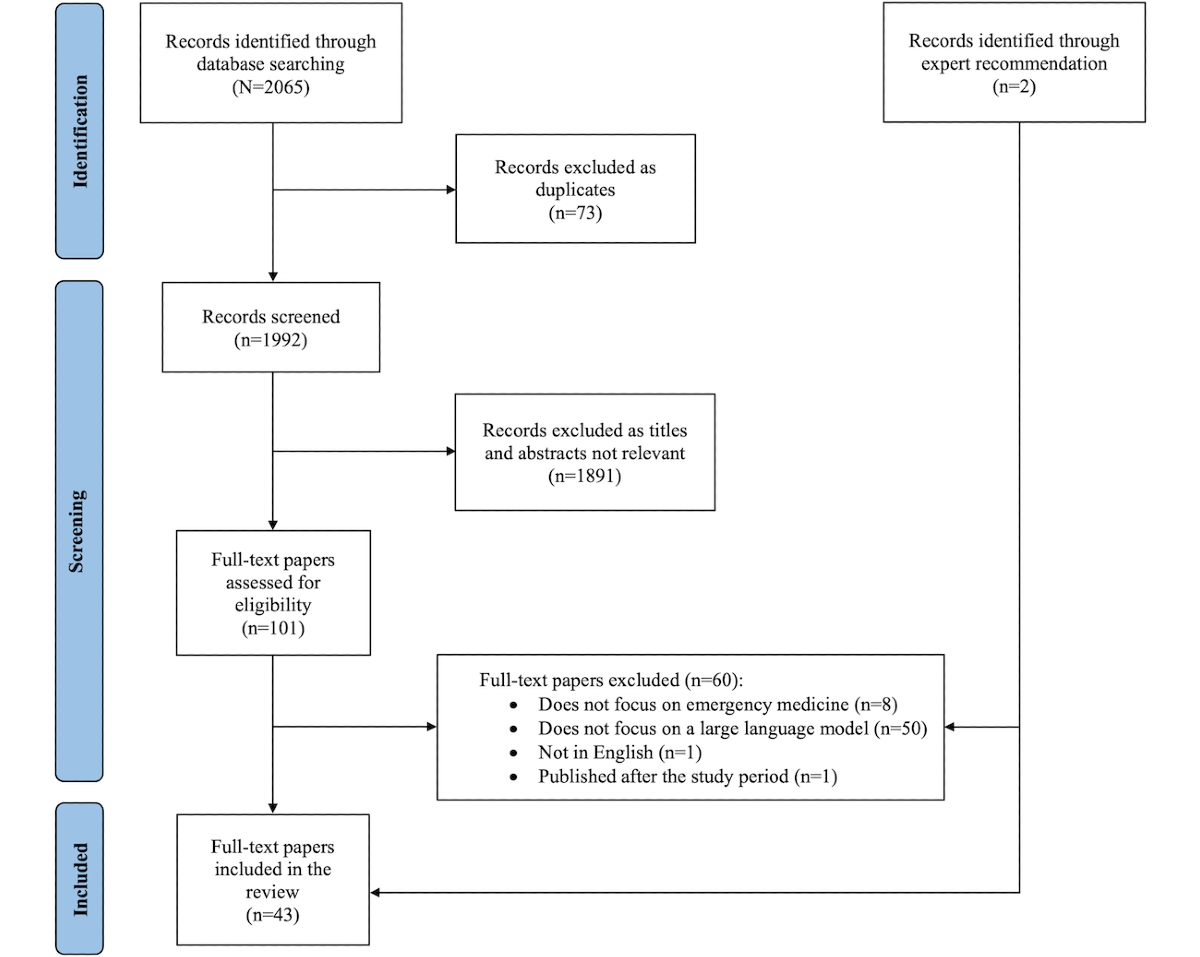
PRISMA (Preferred Reporting Items for Systematic Reviews and Meta-Analyses) flow diagram of search and screening for large language models in emergency medicine.

Two investigators (CP and CR) independently screened 100 abstracts, and the interrater reliability showed substantial agreement (κ=0.75). The remaining abstracts were screened by 1 author (CP), who consulted with a second author as needed for clarification regarding inclusion and exclusion criteria. All papers meeting the initial criteria were independently reviewed in full by 2 authors (CP and CR). Studies determined to meet the eligibility criteria by both reviewers were included in the analysis. Discrepancies were resolved by consensus and with the addition of a third reviewer (NA) if needed. Our initial search strategy identified 2065 papers, of which 73 (3.54%) were duplicates, resulting in 1992 (96.46%) papers for screening (Figure 1). Of the 1992 papers, 1891 (94.93%) were excluded based on the title or abstract. In total, 5.07% (101/1992) of the papers were reviewed in full, and 2.11% (42/1992) of the papers were found to meet the study inclusion criteria. During manuscript review, 2 additional papers were brought to our attention by experts, and 1 of these met the inclusion criteria, bringing the total number of included papers to 43.

### Charting the Data

Data abstraction was independently conducted using a structured form to capture paper details, including the author, year of publication, study type, specific study population, study or paper location, purpose, and main findings. Data to address our primary research question was iteratively abstracted from the papers as our themes emerged, as explained in the subsequent sections.

### Collating, Summarizing, and Reporting the Results

We synthesized and collated the data, performing both a quantitative and qualitative analysis. A descriptive summary of the included studies was created. Then, we used the methodology proposed by Braun and Clarke [12] to conduct a thematic analysis to address our primary research question. Five authors (CP, CR, AC, NA, and RR) independently familiarized themselves with and generated codes for a purposively diverse selection of 10 papers, focusing on content that suggested possible uses for LLMs in EM. The group met to discuss preliminary findings and refine the group’s approach. Individuals then independently aggregated codes into themes. These themes were reviewed and refined as a group. Then, 2 authors (CP and CR) reviewed the remaining manuscripts for any additional themes and data that supported or contradicted our existing themes. These data were used to refine themes through group discussion. Our analysis included a discussion and emphasis on the implications and future research directions for the field, based on the guidance from Levac et al [9].

### Consultation

To ensure our review accurately characterized the available knowledge and that our interpretations of it were correct, we consulted with external emergency physicians with topic expertise in AI. We incorporated feedback as appropriate. For example, we more completely defined LLMs for clarity and included a table describing common models (Table 1). Our findings and recommendations were endorsed by our consultants.

**Table 1:** Large language models reported in the identified literature.

Model	Interface	Model size (parameters)	Developer	Year of release
GPT-3.5 Turbo	ChatGPT	175 billion [13]	OpenAI	2022
GPT-4	ChatGPT	Approximately 1.8 trillion (estimated) [14]	OpenAI	2023
Pathways Language Model	Bard	540 billion [15]	Google AI^a^	2023
Embeddings from Language Model	Full model available	93.6 billion [16]	Allen Institute for AI	2018
Bidirectional Encoder Representations from Transformers	Full model available	110 million and 340 million [17]	Google	2018

^a^AI: artificial intelligence.

## Results

### Overview

Most identified studies (29/43, 67%) were published in 2023. Of the 43 studies, 14 (33%) were conducted in the United States, followed by 6 (14%) in China, 4 (9%) in Australia, 3 (7%) each in Taiwan and France, and 2 (5%) each in Singapore and Korea. Several other individual studies (5/43, 12%) were from various countries (Table 2).

In terms of study type, 40% (17/43) of the papers were methodology studies; 40% (17/43) were case studies; 16% (7/43) were commentaries; and 2% (1/43) each of a case report, qualitative investigation, and retrospective cross-sectional study. In total, 58% (25/43) of these studies addressed the ED setting specifically, followed by 14% (6/43) addressing the prehospital setting and 14% (6/43) addressing other non-ED hospital settings. In total, 7% (3/43) of the studies focused on using LLMs for the public, 5% (2/43) focused on using them for social media analysis, and 2% (1/43) focused on using them for research applications. LLMs used in the reviewed papers (Table 1) included versions of GPT (OpenAI; eg, ChatGPT, GPT-4, and GPT-2), Pathways Language Model (Bard; Google AI), Embeddings from Language Model, XLNet, and BERT (Google; eg, BioBERT, ClinicalBERT, and decoding-enhanced BERT with disentangled information).

We identified four major themes in our analysis: (1) clinical decision-making and support; (2) efficiency, workflow, and information management; (3) risks, ethics, and transparency; and (4) education and communication. Major themes, subthemes, and representative quotations are presented in Table 3.

**Table 2:** Summary of included studies and identified themes (N=43).

Study	Country	Study type	Purpose	Setting and context	Large language models used	Sample size	Themes
Xu et al [18], 2020	France	Methodology	Classification of visits into trauma and nontrauma based on ED^a^ notes	ED	GPT-2 (OpenAI)	16,1930 notes	CDMS^b^ and EWIM^c^
Wang et al [19], 2020	China	Retrospective cross-sectional study	Sentiment analysis of social media posts related to COVID-19	Social media	BERT^d^ (Google)	99,9978 posts	EWIM
Chen et al [20], 2020	Taiwan	Methodology	Diagnosis identification from discharge summaries	Inpatient	BERT and BioBERT	25,8850 discharge diagnoses	EWIM
Chang et al [21], 2020	United States	Methodology	Categorize free-text ED chief complaints	ED	BERT and Embeddings from Language Model	2.1 million adult and pediatric ED visits	CDMS and EWIM
Wang et al [22], 2021	Singapore	Methodology	Summarize EMS^e^ reports for clinical audits	EMS and prehospital	BERT	58,898 ambulance incidents	EWIM
Gil-Jardiné et al [23], 2021	France	Methodology	Classify content of EMS calls during the COVID-19 pandemic	EMS and prehospital	GPT-2	888,469 calls (training), 39,907 calls (validation), and 254,633 calls (application)	EWIM
Shung et al [24], 2021	United States	Methodology	Identify patients with gastrointestinal bleeding from ED triage and ROS data	ED	BERT	7144 cases	CDMS
Tahayori et al [25], 2021	Australia	Methodology	Predict patient disposition from ED triage notes	ED	BERT	249,532 ED encounters	CDMS and EWIM
Kim et al [26], 2021	South Korea	Case study	Assign triage severity to simulated cases	ED	BERT	762 cases	CDMS
Wang et al [27], 2021	China	Methodology	Predict diagnosis and appropriate hospital team from medical record	Prehospital	BERT and ClinicalBERT	198,000 patient records	EWIM
McMaster et al [28], 2021	Australia	Methodology	Identify adverse drug events from discharge summaries	Inpatient	BERT (ClinicalBERT and DeBERTa^f^)	861 discharge summaries	EWIM
Chen et al [29], 2021	Taiwan	Methodology	Classify electronic health record data into disease presentations	ED	BERT	1,040,989 ED visits and 305,897 NHAMCS^g^ samples	EWIM
Drozdov et al [30], 2021	United Kingdom	Methodology	Generate annotations for CXRs^h^ to train model to identify COVID-19 cases	ED	BERT (to generate image annotations)	214,042 CXRs	CDMS
Zhang et al [31], 2022	China	Methodology	Classify EMS cases into disease categories	EMS and prehospital	BERT	3500 records	EWIM
Pease et al [32], 2023	United States	Qualitative investigation	Determine the attitudes of clinicians toward using AI^i^ in suicide screening	ED	N/A^j^	3 clinicians	CDMS and RET^k^
Chae et al [33], 2023	United States	Methodology	Predict ED visits and hospitalizations for patients with heart failure	Prehospital (home health care)	BERT (BioclinicalBERT)	9362 patients	CDMS and RET
Huang et al [34], 2023	United States	Methodology	Predict nonaccidental trauma	ED	BERT	244,326 trajectories (test) and 2,077,852 trajectories (validation)	CDMS
Chen et al [35], 2023	Taiwan	Methodology	Predict critical outcomes from ED data	ED	BERT (comparator)	171,275 ED visits	CDMS
Smith et al [36], 2023	Australia	Case study	Determine model performance on EM^l^ accreditation examination	ED	GPT-3.5 (OpenAI), GPT-4 (OpenAI), Bard-PaLM^m^, Bard-PaLM 2, and Bing (Microsoft Corporation)	240 questions	CDMS, RET, and EC^n^
Gupta et al [37], 2023	United States	Case study	Determine the ability of the model to correctly diagnose simulated cases	ED	ChatGPT	20 cases	CDMS, RET, and EC
Abavisani et al [38], 2023	Iran	Commentary	Potential uses of the model in emergency surgery	Emergency surgery	ChatGPT	N/A	CDMS and RET
Rahman et al [39], 2023	United States	Methodology	Identify cases and patterns in unstructured EMS data	EMS and prehospital	BERT (BioBERT and ClinicaBERT)	40,000 EMS narratives	EWIM
Lam and Au [40], 2023	China	Case study	Evaluate model response to lay questions regarding stroke	General public	ChatGPT	3 questions	EC
Bushuven et al [41], 2023	Germany	Case study	Use of the model to advise parents during pediatric emergencies	General public	ChatGPT and GPT-4	22 cases	CDMS, RET, and EC
Ahn [42], 2023	South Korea	Case study	Use of model to provide a lay-person instruction for cardiopulmonary resuscitation	General public	ChatGPT	3 questions	RET and EC
Preiksaitis et al [43], 2023	United States	Commentary	Potential limitations to using models for clinical charting	General medicine	ChatGPT	N/A	EWIM and RET
Barash et al [44], 2023	Israel	Case study	Use of model to aid radiology referral in the ED	ED	GPT-4	40 cases	CDMS and RET
Dahdah et al [45], 2023	United States	Case study	Use of model to triage based on chief complaints	ED	ChatGPT	30 questions	CDMS and RET
Gottlieb et al [46], 2023	United States	Commentary	Discuss advantages and disadvantages of using the model in research	ED and research	ChatGPT	N/A	RET and EC
Babl and Babl [47], 2023	Australia	Case study	Determine the ability of the model to generate a scientific abstract	Research	ChatGPT	1 abstract	RET and EC
Chen et al [48], 2023	China	Methodology	Use the model to study the functioning of web-based self-organizations	Social media	BERT	47,173 users	EWIM
Bradshaw [49], 2023	United States	Case study	Determine the ability of the model to generate discharge instructions	ED	ChatGPT	1 set of discharge instructions	EWIM and EC
Cheng et al [50], 2023	China	Commentary	Potential uses for the model in surgical management	ED	ChatGPT	N/A	CDMS and EWIM
Rao et al [51], 2023	United States	Case study	Test the model performance in several clinical scenarios	General medicine	ChatGPT	36 clinical vignette	EWIM and EC
Brown et al [52], 2023	Jersey	Case report and commentary	Discuss possible model uses in supporting decision-making and clinical care	ED	ChatGPT	1 case	CDMS and EWIM, RET and EC
Bhattaram et al [53], 2023	India	Case study	The ability of the model to triage clinical scenarios	ED	ChatGPT	5 scenarios	CDMS, RET and EC
Webb [54], 2023	United States	Case study	The ability of the model to be used as a communication skill trainer	ED	ChatGPT-3.5	1 case	RET and EC
Hamed et al [55], 2023	Qatar	Case study	The ability of the model to synthesize clinical practice guidelines for diabetic ketoacidosis	General medicine	ChatGPT	3 guidelines	EWIM and RET
Altamimi et al [56], 2023	Saudi Arabia	Case study	The ability of the model to recommend management in snakebites	ED	ChatGPT	9 questions	CDMS and RET
Gebrael et al [57], 2023	United States	Case study	Predict the disposition of patients with metastatic prostate cancer based on ED documentation	ED	ChatGPT-4	56 patients	CDMS, EWIM, and RET
Sarbay et al [58], 2023	Turkey	Case study	Use of the model for patient triage using clinical scenarios	ED	ChatGPT	50 case scenarios	CDMS, EWIM, and RET
Okada et al [59], 2023	Singapore	Commentary	Discuss possible applications for the model in resuscitation	ED or intensive care unit	GPT-3 and GPT-4	N/A	CDMS, EWIM, and RET
Chenais et al [60], 2023	France	Commentary	Describe the landscape of AI-based applications currently in use in EM	ED	BERT and GPT-2	N/A	CDMS, EWIM, and RET

^a^ED: emergency department.

^b^CDMS: clinical decision-making and support.

^c^EWIM: efficiency, workflow, and information management.

^d^BERT: Bidirectional Encoder Representations from Transformers.

^e^EMS: emergency medical service.

^f^DeBERTa: decoding-enhanced Bidirectional Encoder Representations from Transformers with disentangled information.

^g^NHAMCS: National Hospital Ambulatory Medical Care Survey.

^h^CXR: chest x-ray.

^i^AI: artificial intelligence.

^j^N/A: not applicable.

^k^RET: risks, ethics, and transparency.

^l^EM: emergency medicine.

^m^PaLM: Pathways Language Model.

^n^EC: education and communication.

**Table 3:** Major themes identified, associated subthemes, and representative quotations.

Major theme and subtheme		Representative quotation
**Theme 1: clinical decision-making and support**		
	Prediction	“Machine-learning and natural language processing can be together applied to the ED triage note to predict patient disposition with a high level of accuracy.” [25]
	Treatment recommendations	“An under-explored use of AI in medicine is predicting and synthesizing patient diagnoses, treatment plans, and outcomes.” [51]
	Symptom checking and self-triage	“To our knowledge, this is the first work to investigate the capabilities of ChatGPT and GPT-4 on PALS core cases in the hypothetical scenario that laypersons would use the chatbot for support until EMS arrive.” [41]
	Classification	“In this proof-of-concept study, we demonstrated the process of developing a reliable NER [named-entity recognition] model that could reliably identify clinical entities from unlabeled paramedic free text reports.” [22]
	Triage	“...this preliminary study showed the potential of developing an automatic classification system that directly classifies the KTAS [triage] level and symptoms from the conversations between patients and clinicians.” [26]
	Screening	“We showed that PABLO, a pretrained, domain-adapted outcome forecasting model, can be used to predict both first and recurrent instances of NAT [non-accidental trauma].” [34]
	Differential diagnosis building	“These results suggest that ChatGPT has a high level of accuracy in predicting top differential diagnoses in simulated medical cases.” [37]
	Decision support	“...ChatGPT-4 demonstrates encouraging results as a support tool in the ED. LLMs such as ChatGPT-4 can facilitate appropriate imaging examination selection and improve radiology referral quality.” [44]
	Clinical augmentation	“AI can serve as an adjunct in clinical decision making throughout the entire clinical workflow, from triage to diagnosis to management.” [51]
**Theme 2: efficiency, workflow, and information management**		
	Unstructured data extraction	“The proposed model will provide a method to further extract the unstructured free-text portions in EHRs to obtain an abundance of health data. As we enter the forefront of the artificial intelligence era, NLP deep-learning models are well under development. In our model, all medical free-text data can be transformed into meaningful embeddings, which will enhance medical studies and strengthen doctors’ capabilities.” [20]
	Charting efficiency	“While notes have become more structured and burdensome, the field of data science has rapidly advanced. With such powerful tools available, it seems reasonable to explore their use to automate seemingly mundane tasks such as writing clinical notes. Generative AI models like ChatGPT could be developed to populate notes for patients based on massive amounts of data contained in current EHRs.” [43]
	Summarization or synthesis	“Although ChatGPT demonstrates the potential for the synthesis of clinical guidelines, the presence of multiple recurrent errors and inconsistencies underscores the need for expert human intervention and validation.” [55]
	Pattern identification	“This embedding system can be used as a disease retrieval model, which encodes queries and finds the most relevant patients and diseases. In the retrieval demonstration, the query subject was a 53-year-old female patient who suffered from abdominal pain in the upper right quarter to right flanks for 3 days and noticed dizziness and tarry stool on the day of the interview. Through the retrieval, we obtained the five most similar patients with similar symptoms that were possibly related to different diseases.” [29]
	Workflow efficiency	“Integration of LLMs with existing EHR (with appropriate regulations) could facilitate improved patient outcomes and workflow efficiency.” [51]
**Theme 3: risks, ethics, and transparency**		
	Oversight	“Generally speaking, the Ethics Guideline for Trustworthy AI suggested seven key requirements including human agency and oversight, technical robustness and safety, privacy and data governance, transparency, diversity, nondiscrimination and fairness, environmental and societal well-being, and accountability.” [59]
	Fairness	“[Use of LLMs] could also increase equity by assisting researchers with disabilities such as dyslexia.” [46]
	Ethical and legal responsibilities	“Legal and ethical implications are associated with using AI in clinical practice, particularly regarding privacy and informed consent issues.” [52]
	Reliance on input data	“...data quality can affect the performance of LLMs and NLP techniques applied to the task of extracting and summarizing clinical guidelines.” [55]
	Overreliance	“Overreliance on AI systems and the assumption that they are infallible or less fallible than human judgment–automation bias–can lead to errors.” [52]
	Explainability and transparency	“Creating a clinician-interpretable risk prediction model is essential for clinical adoption and implementation of models because it builds trust in decisionmakers, enables error identification and correction in the model, and facilitates integration into clinical workflows.” [33]
	Bias propagation	“A risk of bias is possible if the initial training data is not representative of the study population. There is a possibility of compounding of bias and error, leading to incorrect assessment.” [53]
	Human bias reduction	“AI tools can offer a near real-time interpretation of medical imaging and clinical decision support and may identify latent patterns that may not be evident to clinicians. While humans are prone to cognitive biases, such as prejudice or fatigue, which can hinder their decision-making process, AI can mitigate these biases and improve accuracy in patient care.” [52]
	Accuracy	“LLMs may not be exposed to the broader range of literature (particularly if studies are located behind paywalls), which may limit the comprehensiveness or accuracy of the data.” [46]
**Theme 4: education and communication**		
	Clinician education	“While LLM performance in medical examinations may initially seem to be little more than a novelty, their ability to generate coherent and well-explained content hints at other potential uses. As a medical education tool they could potentially help generate practice questions, design mock examinations or provide additional explanations for complex concepts.” [36]
	Communication	“Although in its infancy, AI chatbot use has the potential to disrupt how we teach medical students and graduate medical residents communication skills in outpatient and hospital settings.” [54]
	Content generation	“ChatGPT or similar programmes, with careful review of the product by authors, may become a valuable scientific writing tool.” [47]
	Research assistance	“Conversational AI has some clear benefits and disadvantages. As the technology further evolves, it is incumbent on the scientific community to determine how best to incorporate LLMs into the research and publication process with attention to scientific integrity, adherence to ethical principles, and existing copyright laws.” [46]

### Theme 1: Clinical Decision-Making and Support

The first theme we identified is clinical decision-making and support. LLMs have been used or proposed for applications such as providing advice to the public before arrival; aiding in triage as patients arrive at the ED; or augmenting the activities of physicians as they provide care, either through supporting diagnostics or predicting patient resource use.

Several applications focused on advising the public and aiding in symptom checking, self-triage, and occasionally advising first-aid before the arrival of emergency medical services. These included counseling parents during potential pediatric emergencies, recognizing stroke, or providing advice during potential cardiac arrests [40-42]. Wang et al [27] proposed a model that could potentially help patients navigate the complexities of the health care system in China and present to the correct medical setting for the care they need.

Furthermore, LLMs have the potential to efficiently screen patients for important outcomes, such as pediatric patients at risk for nonaccidental trauma, suicide risk, or COVID-19 infection [30,32,34]. These can be implemented based on data in the medical record or as clinical data are obtained in real time.

Early identification of patient risks could help physicians more rapidly identify important diagnoses. Several studies discussed implementations of LLMs that work in conjunction with physicians while caring for patients in the ED [50,51]. Brown et al [52] discuss the potential role of these models in overcoming cognitive biases and reducing errors. These models could be used in developing a differential diagnosis, recommending imaging studies, providing treatment recommendations, or interpreting clinical guidelines [37,44,55,56].

Several studies centered on predicting outcomes such as presentation to the ED, hospitalization, intensive care unit admission, or in-hospital cardiac arrest [25,33,35,57]. Applications of LLMs in the triage process could potentially identify patients who require immediate attention or patients at a high risk of certain diagnoses, such as gastrointestinal bleeding [24,26,53,58,60].

### Theme 2: Efficiency, Workflow, and Information Management

The second theme identified is information management, workflow, and efficiency. LLMs show great promise in increasing the usability of data available in the EHR. Interactions with the EHR take up a substantial amount of physician time, and it is often difficult to identify crucial information during critical times [43]. LLMs could serve a variety of information management functions. They could be used to perform audits for quality improvement purposes, identify potential adverse events such as drug interactions, anticipate and monitor public health emergencies, and assist with information entry during the clinical encounter [19,20,22,23,28,31,39,43,49]. LLMs developed and trained on data from the ED could quickly identify similar patient presentations, recognize patterns, and extract important information from unstructured text [18,20,21,60].

Some authors suggest that LLMs can enhance care throughout the entire EM encounter [30,50-52]. LLMs could potentially be used as digital adjuncts for clinical decision-making because they could generate differentials, predict final diagnoses, offer interpretations of imaging studies, and suggest treatment plans [30,51,52,61]. They may mitigate human cognitive biases and address human factors (eg, time constraints, frequent task switching, high cognitive load, constant interruptions, and decision fatigue) that predispose emergency physicians to error [52].

The flexibility and versatility of the LLMs offer particular benefits to EM practice. The diverse ways in which these models can aid throughout the entire clinical workflow could help physicians process large quantities of complex clinical data, mitigate cognitive biases, and deliver relevant information in a comprehensible format [30,51,52,61]. By streamlining these burdensome tasks, LLMs could help improve the efficiency of care for the high volume of patients the physicians routinely see in the ED.

### Theme 3: Risks, Transparency, and Ethics

Despite the potential for advancement and improvement in the care that EM physicians can provide through the inclusion of LLMs in practice, several issues limit their implementation into practice at this time.

The most often discussed risk, mentioned in 11 (26%) of the 43 papers, is the reliability of model responses and the potential for erroneous results [20,21,28-30,44,51,53,55,56,59]. These output errors often result from inaccuracies in the training data, which are most commonly gathered from the internet and unvetted for reliability. Sources of inaccurate responses may be identified by examining the training material, but other errors due to data noise, mislabeling, or outdated information may be harder to detect [21,28,30,56]. Similarly, biases in training data can be propagated to the model, leading to inaccurate or discriminatory results [51,53,57,60,62]. In medical applications, the consequences of the errors can be significant, and even small errors could lead to adverse outcomes [51].

Understanding and mitigating errors in LLMs is challenging due to issues with transparency and reproducibility of model outputs [52-54,59,62]. Better understanding among clinicians of the algorithms and statistical methods used by LLMs is a suggested method to ensure cautious use [52]. Concentrating on making models more explainable or transparent is another potential approach [62]. However, the degree to which this will be feasible, given the complexity of these models, remains to be determined.

Patient and data privacy is another clearly articulated risk of using these models in the clinical environment [35,52,53]. There are some proposed methodologies using unsupervised methods that can train the models with limited access to sensitive information; however, these require further exploration [35]. Patient attitudes and willingness to allow models access to their health information for training and how to address disclosure of this use have not been extensively discussed. Finally, the legal and ethical implications of using LLM output to guide patient care is an often-mentioned concern [52,53,59]. How the responsibility for patient care decisions is distributed if LLMs are used to guide clinical decisions is yet to be determined.

### Theme 4: Education and Communication

LLMs offer several opportunities for education and communication. First, several papers noted that the successful integration of LLMs into clinical practice will require physicians to understand the underlying algorithms and statistical methods used by these models [52,59]. There is a need for dedicated educational programs on AI in medicine at all levels of medical education to ensure that the solutions developed align with the clinical environment and address the unique challenges of working with clinical data [34,51,63].

In terms of clinical education, several studies have demonstrated reasonable performance of LLMs on standardized tests in medicine, which could indicate the potential for these models to develop study materials [36]. In addition, these models may be able to help physicians communicate with and educate the patients. Dahdah et al [45] used ChatGPT to answer several common medical questions in easy-to-understand language, suggesting the ability to enhance physician responses to patient queries. Webb [54] demonstrated the use of ChatGPT to simulate patient conversation and provide feedback to a physician learning how to break bad news.

Patient education may be facilitated via these models without physician input as well. As discussed in the previous sections, several authors described applications designed to educate patients during emergencies before they arrived in the ED [27,40-42]. Finally, LLMs could be used to aid in knowledge dissemination. Gottleib et al [46] and Babl and Babl [47] describe potential applications for LLMs in research and scientific writing. They highlight potential benefits to individuals who struggle with English or have challenges with writing or knowledge synthesis. In addition, models may be used to translate scientific papers more rapidly. However, the use of these models to generate scientific papers raises concerns regarding the potential for academic dishonesty [46,47].

## Discussion

### Principal Findings

Our review aligns with the growing body of literature emphasizing the great potential for AI in EM, particularly in areas such as time-sensitive decision-making and managing high-volume data [2-5,60]. However, our focus on LLMs and their unique capabilities extends the current understanding of AI applications in EM. Although several specific applications and limitations have been reported and suggested in the literature, our analysis identified 4 major areas of focus for LLMs in EM: clinical decision support, workflow efficiency, risks, ethics, and education. We propose these topics as a framework for understanding emerging implementations of LLMs and as a guide to inform future areas of investigation.

At their core, LLMs and their associated natural language processing techniques offer a way to organize and engage with vast amounts of unstructured text data. Depending on how they are trained and used, they can be operationalized to make predictions or identify patterns, which gives rise to most of our identified applications. Most commercially available LLMs, such as ChatGPT, are trained on massive volumes of text gathered from the internet and then optimized for conversational interaction [64]. This ability to access a breadth of general knowledge and the resulting wide applicability have contributed to the increased use of LLMs by professionals and the public across a variety of fields [65]. As these models become more ubiquitous, there is potential for their use across the care continuum. They could not only support clinical care but also provide an opportunity to offer advice to the public regarding medical concerns. Several papers (3/34, 9%) in our review identified the feasibility of using LLMs to provide first-aid instructions and offer decision support to potential patients seeking care [40-42].

Preliminary work suggests that dedicated training can enhance the ability of these models to make triage recommendations, but prospective implementation has not been tested [27]. LLMs could certainly aid patients in self-triage or with basic medical questions; nevertheless, how this can be effectively and safely implemented needs further exploration, especially with concerns regarding the accuracy of outputs. Possibilities to improve outputs include additional dedicated training of the models to align with the medical and emergency settings to improve their reliability and accuracy. These context-specific models could be equipped with information on the local health care system to help patients identify available resources, schedule appointments, or activate emergency medical services.

In the ED, LLMs could increase workflow efficiency by rapidly synthesizing relevant information from a patient’s medical record, structuring and categorizing chief complaint data, and assigning an emergency severity index level [18,21,26,45,53,58]. In addition, quickly accessing data from the medical record could improve the efficiency and thoroughness of chart review. A model’s ability to identify subtle patterns in data could offer additional diagnostic support by recommending or interpreting laboratory and imaging studies [30,51,52,61]. By facilitating tasks such as information retrieval and synthesis, LLMs could reduce this burden for clinicians and minimize errors due to buried or disorganized data, potentially contributing to workflow efficiency. Furthermore, they may counteract human cognitive biases and fatigue when used to support clinical decisions [52]. Although some studies have demonstrated reasonable accuracy on focused use cases, further validation of any of these applications across diverse settings and patient populations is required. Thoughtful integration of LLMs has the potential to revolutionize EM by providing clinical decision support, improving situational awareness, and increasing productivity.

However, barriers to seamless implementation exist. As noted by several authors, erroneous outputs remain a concern, given the dependence on training data [28-30,35,51,53,55,56,59]. Information surrounding the most publicly available LLMs today is obscured across three important layers: (1) the underlying training data used—commonly reported to be publicly available data on the internet and from third-party licensed data sets, (2) the underlying architecture of the model—whose exact mechanisms are not always easy to discern, and (3) the intricacies of human-led fine-tuning—often done at the end of development to provide guardrails for output. These layers of obscurity make it difficult to troubleshoot the cause of any single erroneous output.

Regarding privacy and data rights, it is imperative to discuss and implement privacy-preserving methods for patient data. The use of techniques such as data anonymization, differential privacy, and federated learning are instrumental in safeguarding patient information. Data anonymization involves removing or modifying personal identifiers to prevent the association of data with individual patients. Differential privacy introduces randomness into the data or queries to ensure individual data points cannot be isolated [66]. Federated learning enables models to be trained against multiple decentralized devices or servers holding local data samples without exchanging them, thus enhancing privacy [67]. The specific ways in which LLMs will interface with other hospital information systems, such as the EHR, need further exploration, and careful integration is critical to address privacy concerns, especially given the sensitive nature of health care data.

Moreover, the ongoing discussions about the information used in these models underscore the need for continuous scrutiny [52,53,59]. In addition to privacy, the legal and ethical implications of AI-assisted health care require further exploration to establish robust oversight and accountability structures. Without a commitment to explainability and transparency, the use of *black box* LLMs may encounter resistance from clinicians.

Our review reveals several opportunities for future exploration and research. Perhaps the most important is effectively identifying problems that are best solved using LLMs in EM. Our review outlines several immediate areas of potential exploration, including improved communication, translation, and summarization of highly detailed and domain-specific knowledge for providers and patients, but further exploration and prospective validation of specific use cases is required. We expect the potential use cases in EM to grow as LLMs become increasingly complex and develop emergent properties–actions that are not explicitly programmed or anticipated. To bridge the *AI chasm* between innovations in the research realm and widespread adoption, these applications should be identified with significant input from providers in the clinical space who can uniquely identify areas of potential benefit. To accomplish this, a better understanding of the abilities and limitations of LLMs among physicians is needed to optimize their best use and ensure they are effectively implemented, and AI literacy is increasingly described as an essential competency for physicians [68]. We encourage the development of curricula and training programs designed for emergency physicians.

Given the black-box nature of LLMs, standardized frameworks and metrics for evaluation that are specific to health care use cases are needed to evaluate their performance and implementation effectively. These frameworks should encompass an understanding of both the technical capabilities and constraints of a model, along with the human interaction aspects that affect its use. A crucial part of this assessment involves comparing the performance of LLMs to human proficiency, determining whether the objective is to replace or enhance tasks currently carried out by health care professionals. Thorough testing of models in real time, real-world scenarios is imperative before their deployment. The selection of patient- or provider-focused outcomes is essential, and the effectiveness of models should not be evaluated in isolation. Instead, it is crucial to assess the combined performance of the provider and AI system to ensure that models are effective and practical in real-world settings. Implementing and validating solutions should occur across diverse populations and care environments, with particular focus on cohorts underrepresented in the training data to mitigate potential harm from model biases [69]. Provider perspectives are essential, but equally important are patient perspectives about the use of LLMs in medicine. Impacts on physician-patient communication, patient concerns surrounding privacy, and attitudes toward AI-generated recommendations must be further explored. Collaboration between all relevant stakeholders who develop or will be impacted by LLMs for clinical medicine is essential for developing models that can be used effectively, equitably, and safely.

### Limitations

This scoping review has some limitations worth noting. First, we restricted our search to papers published after 2018, when LLMs first emerged. While this captures the current era of LLMs, earlier works relevant to natural language processing in EM may have been overlooked. In addition, despite searching 4 databases and consulting a medical librarian on the search strategy, some pertinent studies may have been missed, and given the rapidly evolving nature of this research area, there are certainly more studies that have emerged since our literature search [70]. However, our review establishes an initial foundation that can be built upon as the field continues to grow. Finally, in an effort to be maximally inclusive in our review, we did not include or exclude papers based on the quality of their evidence. Similarly, we did not make any quality determinations of our included studies. High-quality studies are required to make any determination regarding the efficacy of LLMs for the applications we described, and our review hopefully provides a framework to design these investigations.

### Conclusions

This review underscores the transformative potential of LLMs in enhancing the delivery of emergency care. By leveraging their ability to process vast amounts of data rapidly, LLMs offer unprecedented opportunities to improve decision-making speed and accuracy, a critical component in the high-stakes, fast-paced EM environment. From the identified themes, it is evident that LLMs have the potential to revolutionize various aspects of emergency care, highlighting their versatility and the breadth of their applicability.

From the theme of clinical decision-making and support, LLMs can augment the diagnostic process, support differential diagnosis, and aid in the efficient allocation of resources. In the domain of efficiency, workflow, and information management, LLMs have shown promise in enhancing operational efficiencies, reducing the cognitive load on clinicians, and streamlining patient care processes. Regarding risks, ethics, and transparency, the review illuminates the need for meticulous attention to the accuracy, bias, and ethical considerations inherent in deploying LLMs in a clinical setting. Finally, in the realm of education and communication, LLMs’ potential to facilitate learning and improve patient and provider communication signifies a paradigm shift in medical education and engagement.

The most urgent research need identified in this review is the development of robust, evidence-based frameworks for evaluating the clinical efficacy of LLMs in EM; addressing ethical concerns; ensuring data privacy; and mitigating potential biases in model outputs. There is a critical need for prospective studies that validate the utility of LLMs in real-world emergency care settings and explore the optimization of these models for specific clinical tasks. Furthermore, research should focus on understanding the best practices for integrating LLMs into the existing health care workflows without disrupting the clinician-patient relationship.

The successful integration of LLMs into EM necessitates a multidisciplinary approach involving clinicians, computer scientists, ethicists, patients, and policy makers. Collaborative efforts are essential to navigate the challenges of implementing AI technologies in health care, ensuring LLMs complement the clinical judgment of EM professionals and align with the overarching goal of improving patient care. The judicious application of LLMs has the potential to fundamentally redefine much of EM practice, ushering in a future where care is more accurate, efficient, and responsive to the needs of patients. Furthermore, by reducing the many burdens that currently encumber clinicians, these technologies hold the promise of restoring and deepening the invaluable human connections between physicians and their patients.

## Appendix

Multimedia Appendix 1 Literature review search strategy.

Multimedia Appendix 2 PRISMA-ScR checklist.

## Abbreviations

AI: artificial intelligence
BERT: Bidirectional Encoder Representations from Transformers
ED: emergency department
EHR: electronic health record
EM: emergency medicine
LLM: large language model
MeSH: Medical Subject Headings
PRISMA-ScR: Preferred Reporting Items for Systematic Reviews and Meta-Analyses extension for Scoping Reviews
