# Supplementary material for: The Role of Large Language Models in Transforming Emergency Medicine: Scoping Review
Source: JMIR Med Inform. 2024 May 10;12:e53787. doi: 10.2196/53787 (PMC11127144; doi:10.2196/53787)
Supplement: Multimedia Appendix 1 [file medinform_v12i1e53787_app1.docx]

**Appendix 1: Literature Review Search Strategy**

(All limited to after 2018)

OVID MEDLINE

(large language model* OR language neural network* OR generative ai OR generative artificial intelligence OR foundation model* OR medpalm OR gpt OR chatgpt OR pre trained model* OR pretrained model* OR conversational ai OR transformer model* OR deep learning language model* OR language model* OR xlnet model* OR bert model* OR language generation model*).ti,ab,kf

AND

(immediate care OR emergency OR para medic* OR paramedic* OR acute care OR unscheduled OR emergent care OR emergicenter?).ti,ab,kf OR exp Emergency Medicine/ OR (prehospital care OR pre hospital care).ti,ab,kf OR exp Emergency Medical Technicians/ OR Emergency Medical Services/ OR Emergency Service, Hospital/ OR exp Emergency Medical Dispatch/ OR (911 dispatch OR "9 1 1" dispatch).ti,ab,kf OR exp Emergency Medical Service Communication Systems/

Web of Science

TS= ((large language model* OR language neural network* OR generative ai OR generative artificial intelligence OR foundation model* OR medpalm OR gpt OR chatgpt OR pre trained model* OR pretrained model* OR conversational ai OR transformer model* OR deep learning language model* OR language model* OR xlnet model* OR bert model* OR language generation model*))

AND

TS= ((immediate care OR emergency OR para medic* OR paramedic* OR acute care OR unscheduled OR emergent care OR emergicenter? OR prehospital care OR pre hospital care OR 911 dispatch OR "9 1 1" dispatch))

Embase

('large language model':ti,ab,kw OR 'language neural network':ti,ab,kw OR 'generative ai':ti,ab,kw OR 'generative artificial intelligence':ti,ab,kw OR 'foundation model':ti,ab,kw OR medpalm:ti,ab,kw OR gpt:ti,ab,kw OR chatgpt:ti,ab,kw OR 'pre trained model':ti,ab,kw OR 'pretrained model':ti,ab,kw OR 'conversational ai':ti,ab,kw OR 'transformer model':ti,ab,kw OR 'deep learning language model':ti,ab,kw OR 'language model':ti,ab,kw OR 'xlnet model':ti,ab,kw OR 'bert model':ti,ab,kw OR 'language generation model':ti,ab,kw)

AND

('immediate care':ti,ab,kw OR emergency:ti,ab,kw OR 'para medic':ti,ab,kw OR paramedic:ti,ab,kw OR 'acute care':ti,ab,kw OR unscheduled:ti,ab,kw OR 'emergent care':ti,ab,kw OR emergicenter:ti,ab,kw OR 'prehospital care':ti,ab,kw OR 'pre hospital care':ti,ab,kw OR '911 dispatch':ti,ab,kw OR '9 1 1 dispatch':ti,ab,kw)

OR

exp ‘emergency health service’/

Google Scholar

("large language model" OR "language neural network" OR "generative ai" OR "foundation model" OR medpalm OR chatgpt OR "pretrained model") AND ("immediate care" OR emergency OR paramedic OR "acute care" OR unscheduled OR "emergent care" OR emergicenter)

("large language model" OR "language neural network" OR "generative ai" OR "foundation model" OR medpalm OR chatgpt OR "pretrained model") AND ("prehospital care" OR "pre hospital care" OR "911 dispatch" OR "9 1 1 dispatch")

("conversational ai" OR "transformer model" OR "deep learning language model" OR "xlnet model" OR "bert model" OR "language generation model") AND ("immediate care" OR emergency OR paramedic OR "acute care" OR unscheduled OR "emergent care" OR emergicenter)

("conversational ai" OR "transformer model" OR "deep learning language model" OR "xlnet model" OR "bert model" OR "language generation model") AND ("prehospital care" OR "pre hospital care" OR "911 dispatch" OR "9 1 1 dispatch")
